# p53 deficiency mediates cisplatin resistance by upregulating RRM2 and crotonylation of RRM2^K283^ through the downregulation of SIRT7

**DOI:** 10.3389/fmolb.2024.1423594

**Published:** 2024-06-04

**Authors:** Liangjie Sun, Yi Li, Meng Wang, Lan Luo, Ruiqing Sun, Yang Chen, Yan Bai, Chong Ding, Yixiang Wang

**Affiliations:** ^1^ Central Laboratory, Peking University School and Hospital of Stomatology, National Center for Stomatology, National Clinical Research Center for Oral Diseases, National Engineering Research Center of Oral Biomaterials and Digital Medical Devices, Beijing Key Laboratory of Digital Stomatology, NHC Key Laboratory of Digital Stomatology, NMPA Key Laboratory for Dental Materials, Beijing, China; ^2^ Shanxi Medical University School and Hospital of Stomatology, Shanxi Province Key Laboratory of Oral Diseases Prevention and New Materials, Taiyuan, China; ^3^ Department of Pediatric Dentistry, Peking University School and Hospital of Stomatology, Beijing, China

**Keywords:** p53 deficiency, SIRT7, RRM2, crotonylation, cisplatin resistance

## Abstract

p53 deficiency plays a crucial role in chemotherapy resistance through various biological events, including posttranslational modifications (PTMs). Recently, lysine crotonylation (Kcr) has been shown to play a vital role in cancer progression. However, the global p53-regulated crotonylome and the function of these altered Kcr proteins after p53 deficiency remain unclear. In this study, we used a SILAC-based quantitative crotonylome to identify 3,520 Kcr in 1924 crotonylated proteins in response to p53 knockout. We found that increased crotonylation of RRM2 at K283 (RRM2^K283Cr^) in the presence of p53 deficiency promoted HCT116 cell resistance to cisplatin. We discovered that SIRT7 could be the decrotonylase of RRM2 and was downregulated after p53 knockout, resulting in increased RRM2^K283Cr^. Mechanistically, p53 deficiency inhibited cell apoptosis by upregulating RRM2 protein expression and RRM2^K283Cr^-mediated cleaved-PARP1 and cleaved-caspase3 expression, and SIRT7 was downregulated to upregulate crotonylation of RRM2 upon p53 deficiency. In conclusion, our results indicated that p53 deficiency plays a malignant role in colon cancer resistance to cisplatin therapy by regulating RRM2 protein and RRM2^K283Cr^ expression. Our findings provide a novel therapeutic target against p53-deficient cancer.

## Introduction

Colorectal cancer ranks as the third most frequently diagnosed cancer globally. With an estimated global incidence of approximately 1.9 million cases and mortality rates reaching nearly 940,000 individuals annually, colorectal cancer is the second leading cause of cancer-related death (Sung et al., 2021). The main clinical treatment for colon cancer is surgical resection combined with chemotherapy (van der Stok et al., 2017). However, advancements in colon cancer chemotherapeutics have been relatively restricted over the past 50 years, with initial treatments such as 5-fluorouracil (5-FU) and cisplatin remaining fundamental to chemotherapeutic protocols and drug resistance.

p53, encoded by the TP53 gene, is the most frequently mutated gene in human tumors and plays a significant role in cell proliferation and preventing cancer conformation (Levine, 1997). Studies have shown that p53 inactivation results in resistance to cisplatin in astrocytic glioma cells (Xu et al., 2005) and 833K testis tumor cell lines (Köberle and Schoch, 2021). Colorectal cancer has the highest occurrence of p53 mutations, with 53% in all colon cancer samples and approximately 70% in recurrent samples (Gao et al., 2022), resulting in the loss of tumor-suppressing function, which endows tumor cells with an advantage by preventing intrinsic tumor suppressive responses such as senescence and apoptosis in response to chemotherapy (Hu et al., 2021). Cisplatin resistance in colorectal cancer is attributed to changes in the p53-mediated DNA damage response and the loss of functional futile mismatch repair (Köberle and Schoch, 2021). Although the impacts of p53 inactivation on cisplatin resistance have been studied in various tumors, the underlying mechanisms are still unclear.

Ribonucleotide reductase (RNR) plays a crucial role in DNA synthesis because it is the sole enzyme responsible for catalyzing the *de novo* formation of deoxyribonucleotides. RNR has two types of subunits that come together to form a tetramer (α2β2 complex) and carry out catalytic activity (Uhlin and Eklund, 1994). RRM2, a small subunit of the RNR, provides deoxyribonucleoside triphosphates (dNTPs) for the replication and repair of nuclear DNA during the S phase of cell proliferation (Zuo et al., 2022). Studies have shown that the RRM2 gene and its promoter region are highly amplified in cancer, resulting in elevated transcription levels, and RRM2 is recognized as a promoter of tumor growth and drug resistance (Zuo et al., 2022). Liu et al. (2019) reported increased RRM2 mRNA expression in peripheral blood samples from patients resistant to imatinib. Therefore, RRM2 has the potential to serve as a biomarker and has already been used as a therapeutic target for chemotherapy. As one of the RRM2 inhibitors, 3-aminopyridine-2-carboxaldehyde thiosemicarbazone (3-AP) has the potential to restore sensitivity to platinum-based treatment in cases of platinum-resistant ovarian cancer (Kunos et al., 2012). However, whether RRM2 plays a role in cisplatin resistance in colon cancer and by what mechanism are still unknown.

In 2011, histone lysine crotonylation was initially recognized as a novel form of posttranslational modification (Tan et al., 2011). Lysine crotonylation (Kcr) is regulated by a set of enzymes, such as crotonyltransferase, decrotonylase and the substrate crotonyl-CoA, an intermediate metabolite derived from fatty acid metabolism, is catalyzed by acyl-CoA synthetase short-chain family member 2 (ACSS2) (Xu et al., 2017). Crotonyltransferases includes p300/CBP, MYST and GNAT (Hou et al., 2021a). Decrotonylase includes HDAC and sirtuin family members (Wan et al., 2019). Recently, significant advancements have been achieved in the exploration of both histone and nonhistone crotonylation in the contexts of infectious diseases, kidney diseases, cardiovascular diseases, metabolic diseases, spermatogenesis disorders and cancer (Yang et al., 2023). Crotonylation is closely related to the occurrence and progression of colon cancer. The level of nonhistone ENO1-Kcr is elevated in colorectal cancer tissues, and ENO1^K420Cr^ has been shown to stimulate the growth, migration and invasion of colorectal cancer cells *in vitro* (Hou et al., 2021b). Qu (Qu et al., 2023) et al. reported that p300/CBP-mediated Kcr plays a role in the induction of hypoxia by ATX in SW480 cells. Liao (Liao et al., 2022) et al. reported a decrease in the level of H3K27cr, which may be facilitated by SIRT6 during DNA damage in colon cancer. However, whether p53 is capable of regulating the crotonylation of nonhistone proteins, subsequently contributing to cisplatin resistance, as well as the identity of the regulators and the specific crotonylation sites on p53-targeted proteins, remains to be elucidated.

In this study, we used a quantitative proteomics approach to comprehensively analyze the response of the crotonylome to p53 knockout (KO) in HCT116 cells. Among the 3,502 Kcr sites identified in 1924 crotonylated proteins, we focused on investigating the functional significance of p53-upregulated K283Cr in RRM2 (RRM2^K283Cr^), which is regulated by SIRT7. In particular, our findings demonstrated the crucial role of RRM2^K283Cr^ in mediating cisplatin resistance by modulating cell apoptosis in p53-deficient HCT116 cells.

## Materials and methods

### Cell lines and culture

The human CRC cell line HCT116 (p53^+/+^) was obtained from the American Type Culture Collection (CCL-247, ATCC, Manassas, Virginia, USA). The p53 knockout HCT116 cell line (p53^−/−^) was kindly provided by Dr. Bert Vogelstein (John Hopkins University of Medicine). The p53^+/+^ and p53^−/−^ HCT116 cell lines were cultured in Dulbecco’s modified Eagle’s medium (DMEM; Gibco, California, USA) supplemented with 10% fetal bovine serum (FBS; PAN-seratech, Aidenbach, Germany) and 1% penicillin/streptomycin (Solarbio) in a 5% CO_2_ incubator.

### Antibody and reagents

Primary antibodies against cleaved-PARP1 (A19612), HDAC2 (A19626), HDAC3 (A2139), HDAC6 (A11259), SIRT1 (A11267), RRM2 (A3424) and GAPDH (A19056) were obtained from ABclonal (Wuhan, China). Antibodies against HDAC1 (380787), HDAC5 (344279), and SIRT6 (20049-6C9), caspase-3 (R23315), and PARP1 (380451) were purchased from Zenbio (Chengdu, China). Antibodies against HDAC7 (AF2659), HDAC10 (AF26650), SIRT2 (AF2383) and SIRT7 (AF7986) were purchased from Beyotime (Shanghai, China). Antibodies against cleaved-caspase3 (9,664) were obtained from Cell Signaling Technology (Massachusetts, USA). An anti-p53 antibody (ab1101) was purchased from Abcam (Cambridge, UK). An anti-Pan-Kcr (PTM-501) was purchased from PTM-BioLab (Hangzhou, Zhejiang, China). Cisplatin was purchased from YUNNAN Phytopharmaceutical Co., Ltd.

### siRNA, plasmids information

siRNAs were designed and synthesized by Ribo biotech company (https://www.ribobio.com/). The genOFF st-h-RRM2 (siRRM2, Cat: stB0003324A) was purchased from Ribo Biotech Company. The sequences of the siRRM2 and corresponding negative control siNC (Cat: siN000001-1-5) is confidential by the company owing to business secret. OE SIRT7 plasmid (pip-SIRT7-Flag, Cat number SP-1970), gifted from BRICS plasmid resource sharing platform (http://www.brics.ac.cn/). OE Empty Vector plasmid is derived from the pip-SIRT7-Flag plasmid and constructed by our group. Briefly, the pip-SIRT7-Flag plasmid was digested by BamHI and PstI. After the major part of the coding sequence of SIRT7 was cut off, the vector large fragment was purified, and self-cyclizated by T4 DNA ligase, then transformed into *E. coli* competent cells. After screening of recombinant transformants, after the large fragment was filled was the OE Empty Vector plasmid was constructed and then used as the control of the pip-SIRT7-Flag plasmid.

#### Cell culture and plasmid/siRNA transfection

HCT116 p53^+/+^ and HCT116 p53^−/−^ cells were seeded in a 6-well plate at a density of 1×10^6^ cells/well and allowed to proliferate until approximately 60% confluence. Plasmid and siRNA transfection was carried out with Lipo8000^TM^ (Beyotime, Shanghai, China) transfection reagent according to the manufacturer’s recommendations. Overexpression and downregulation of the indicated genes were detected by western blot at 48 h posttransfection.

#### Crotonylome analysis

Crotonylomics reasearch was done by PTM-BioLab Company (Hangzhou, Zhejiang, China). Briefly, the crontonylated protein lysis samples of HCT116 p53^+/+^ and HCT116 p53^−/−^ cells were enriched by anti-pan-Kcr beads (Cat: PTM503, PTM-BioLab) at 4°C overnight with gentle shaking, and then the bound peptides were subjected to MS/MS analysis, protein database searching, gene ontology (GO) annotation Encyclopedia of Genes and Genomes (KEGG) pathway, protein domains subcellular localization prediction, and protein‒protein interaction analyses by using SwissProt, UniProt-GOA, Encyclopedia of Genes and Genomes (KEGG), InterPro, Wolfpsort and STRING databases, respectively. Threshold value was set based on GO and KEGG with a two-tailed Fisher’s exact test results, *p*-value < 0.05 and fold enrichment>1.5 and mapping (at least two proteins are in this function/pathway.) >1 was considered significant. A confidence score ≥0.7 was considered to indicate high confidence.

### RNA extraction, reverse transcription and qPCR analysis

RNA extraction and cDNA synthesis were carried out using the TRIzol reagent (Invitrogen, Carlsbad, CA, USA) and Reverse Transcription kit (Promega, Madison, WI, USA) respectively, according to the manufacturer’s instructions. Gene amplification was performed using qPCR with the SYBR Green master mix (ABclonal) on a qPCR instrument (Bio-Rad, Hercules, CA, United States). The thermocycling protocol of qRT-PCR procedure were set as follows: an initial denaturation at 95°C for 7 min, 40 cycles, including denaturation at 95°C for 5 s, annealing and elongation at 60°C for 30 s, 60°C for 30 s and 20°C for 10 s. The Ct data analysis was performed using the 2^−ΔΔCt^ methods. GAPDH served as an internal control. Primer sequences were listed as follows:

GAPDH (forward: 5′-GGT​CAC​CAG​GGC​TGC​TTT​TA-3’; reverse: 5′-GGA​TCT​CGC​TCC​TGG​AAG​ATG-3′), RRM2 (forward: 5’ -GTG​GAG​CGA​TTT​AGC​CAA​GAA-3’; reverse: 5′-CAC​AAG​GCA​TCG​TTT​CAA​TGG-3′), ATP7A (forward: 5′-TGT​GTG​CAG​TCT​ATT​GAG​GGT-3’; reverse: 5′-TGA​CAA​GGT​AGC​ATC​AAA​TCC​C-3′), ATP7B (forward: 5′-GCC​AGC​ATT​GCA​GAA​GGA​AAG-3’; reverse: 5′-TGA​TAA​GTG​ATG​ACG​GCC​TCT-3′), MUC16 (forward: 5′-GGA​GCA​CAC​GCT​AGT​TCA​GAA-3’; reverse: 5′- GGT​CTC​TAT​TGA​GGG​GAA​GGT-3′).

#### Western blot

The cells were lysed in cell lysis buffer (Cat: P0013, Beyotime) supplemented with protease and phosphatase inhibitor cocktail (Cat: P1046, Beyotime). Twenty micrograms of protein extracts were separated by 12.5% sodium dodecyl sulfate‒polyacrylamide gel electrophoresis (SDS-PAGE). The proteins were transferred onto polyvinylidene difluoride membranes (PVDF; Millipore, USA), blocked with 5% nonfat milk for 1 h at room temperature, and then incubated with diluted primary antibodies (1:1000 dilution) overnight at 4°C. The membrane was then washed with Tris-buffered saline tween-20 (TBST, 20 mM Tris, 150 mM NaCl, 0.1% Tween-20, PH 7.4) three times and probed with HRP-conjugated secondary antibodies (1:10000) for 1 h at room temperature. The proteins were visualized with a western blotting detection system (e-BLOT, China).

#### Co-immunoprecipitation (co-IP) assay

HCT116 cells were lysed by adding 500 μL of lysis buffer with proteinase inhibitors to 10 cm plates and then centrifuged at 12,000 × g for 15 min to obtain the supernatant. The supernatant was quantified by the BCA method. Protein A magnetic beads (Cat: P2175S, Beyotime) were preincubated with diluted antibodies on a rotary mixer at room temperature for 1 h. Equal amounts of cell lysates obtained in the previous step were incubated with antibody-coated magnetic beads overnight at 4°C. After washing with lysis buffer containing protease inhibitors three times, the beads were eluted with SDS‒PAGE sample loading buffer (1×) and boiled at 95°C for 5 min. The precipitated protein complexes were subjected to western blot after the magnetic beads were removed.

#### Cell viability assay

HCT116 cells were seeded into 96-well plates at a density of 10,000 cells/100 μL of medium per well and allowed to incubate overnight in an incubator. On the following day, the cells were cultured with different concentrations of cisplatin (0, 5, 15, 30, or 60 μM) for 24 h. Cell Counting Kit-8 (CCK-8; Dojindo, Shanghai, China) reagents were added to each well, and the plates were incubated for 1 h. The absorbance at 450 nm was measured by using a microplate reader (Bio-Tek, Elx808^TM^, USA). The cell viability rate was calculated by the formula [(As-Ab)/(Ac-Ab)]×100%, where As is the absorbance of the cisplatin treatment well; AC is the absorbance of the control well; and Ab is the absorbance of the blank well. The IC50 values were determined by GraphPad Prism 10 software. Each experiment was performed in duplicate.

#### Clonogenic assays

Cells were inoculated into 6-well plates at 800 cells/2 mL complete medium/well and cultured overnight. The next day, the cells were treated with/without 25 μM cisplatin-containing medium for 24 h. The medium was replaced with drug-free medium, and the cells were allowed to form colonies for another 2 weeks. The colonies were fixed and stained with 0.1% crystal violet (Cat: G1062, Solarbio) for 15 min. Thereafter, the colony numbers were recorded and counted by ImageJ software (Java 13.0.6 (64-bit). Each experiment was performed in duplicate.

#### Apoptosis detection assay

An apoptosis detection assay was performed by using a FITC-Annexin V/PI Apoptosis Detection Kit (Cat: RK05875, ABclonal). HCT116 cells were seeded in 6-well plates at 5×10^5^ and treated with cisplatin for 24 h. The cells were harvested by 0.25% trypsin with EDTA. The cells were washed with precooled PBS two times and resuspended in 1× binding buffer. According to the manufacturer’s protocol, FITC-conjugated Annexin V antibody and PI staining solution were added to the suspension, which was then incubated for 15 min at room temperature in the dark. Finally, the samples were analyzed by using a flow cytometer. Each experiment was performed three times.

#### Subcutaneous xenograft and therapy assays

The animal experiments were approved by Institutional Animal Care and Use Committee of Peking University Health Center (DLASBD0197). Six-week-old male BALB/c nude mice were obtained from Vital Laboratory Animal Co., Ltd. (Beijing, China). The animal experiment was carried out in the animal laboratory of Peking University School of Stomatology. The feeding environment is a barrier SPF environment with 20°C–26°C, 40%–70% humidity, 12 h light and 12 h dark, and all mice are freedom from hunger, thirst, pain, injure, fear and distress, freedom to express normal behavior. All mice were randomly divided into two groups. p53^+/+^ and p53^−/−^ HCT116 cells were trypsinized and resuspended in precooled PBS, and the cell density was adjusted to 5×10^7^ cells/mL. One hundred microliters of cell suspension was injected subcutaneously into the back of nude mice (five mice per group). At 3 days after tumor cell inoculation, the mice were intraperitoneally injected with cisplatin once every other day, and the tumor size and weight were measured every 2 days. The volume of the tumor was calculated by the formula below: volume = 0.5×length×width^2^ (mm^3^). PBS served as a negative control. Mice bearing tumor larger than 1000 mm^3^ were judged dead, and were humanely euthanized by excessive CO_2_ inhalation (100% CO_2_ was induced at a flow of 6 L/min) for euthanasia. The animal experiment took 3 weeks in total. Animals with tumors that do not meet this criterion are euthanized uniformly at the end of the experiment. At the same time, we followed the principle that tumors should not reach a location that significantly affects the normal function of the animals, or cause pain due to tumor growth, and that weight loss in animals should not exceed 20% of their normal weight.

#### Histology and immunohistochemistry analysis

The tumors were fixed in formalin, embedded in paraffin, and then cut into 4-μm-thick slides. Subsequently, the slides were deparaffinized in xylene, followed by rehydration through an ethanol gradient. The sections were subjected to antigen retrieval by heating in a pressure cooker at 120°C and 100 kPa in 0.01 mol/L sodium citrate (pH 6.0) for 5 min. To remove endogenous enzyme and oxidation-reducing activity in the tissues, the slides were stored in a 3% hydrogen peroxide solution at room temperature for 20 min in the dark and then blocked with 10% goat serum for nonspecific binding for 60 min at 37°C. Each slide was then incubated with primary antibodies overnight at 4°C and 100% humidity. The next day, horseradish peroxidase (HRP)-labeled secondary antibody (ZSGB-BIO, Beijing, China) was applied and incubated at 37°C for 60 min, followed by color development using 3,3′-diaminobenzidine (DAB, ZLI-9019, ZSGB-BIO). After counterstaining with hematoxylin, the sections were observed and recorded using a microscope with an Olympus DP controller (Japan). The immunoreactivity was calculated using ImageJ software based on three representative fields.

### Statistical analysis

Statistical analysis was performed using GraphPad Prism 10.0 software to assess differences between the experimental groups. An unpaired *t*-test was used to compare continuous variables. For comparisons involving more than two groups, the results were statistically analyzed using one-way ANOVA followed by Bonferroni’s *post hoc* test. *p* < 0.05 was considered statistically significant.

## Results

### p53 deficiency enhanced cisplatin resistance in HCT116 cells

**FIGURE 1:**
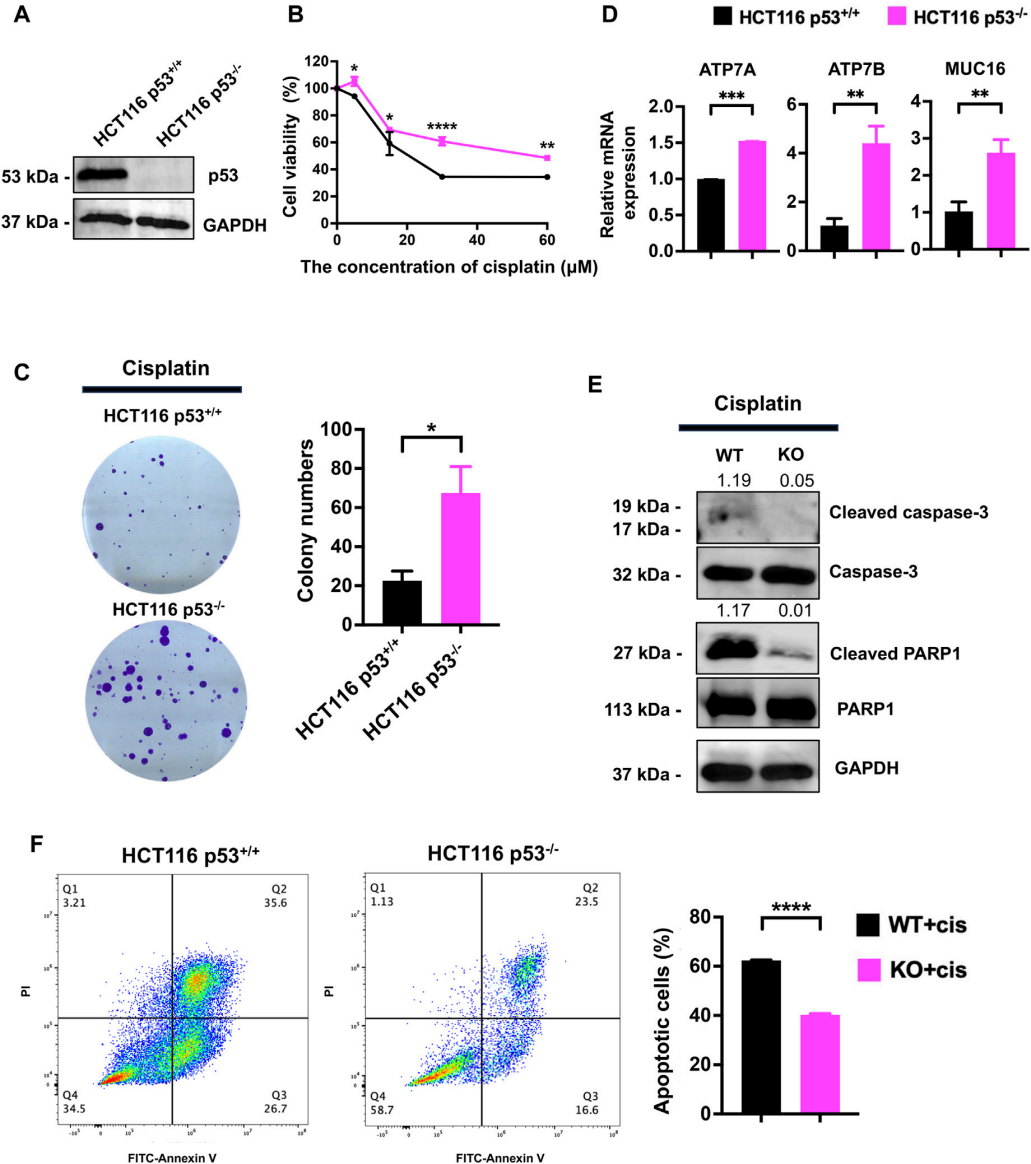
p53 deficiency increases the resistance of HCT116 cells to cisplatin. **(A)** Western blot was performed to assess the expression of p53 in HCT116 p53^+/+^ and HCT116 p53^−/−^ cells. **(B)** The CCK-8 assay was used to assess the viability of cells treated with cisplatin at concentrations of 0, 5, 15, 30, and 60 μM. **(C)** The cell survival and colony formation assay was used to assess the proliferation capability of cells treated with 10 μM cisplatin. **(D, E)** HCT116 p53^+/+^ and HCT116 p53^−/−^ cells were treated with 25 μM cisplatin for 48 h. qPCR was performed to detect cisplatin resistance genes ATP7A, ATP7B and MUC16 expression. Western blot was used to detect the expression of cleaved-caspase3, cleaved-PARP1, p53 and GAPDH. The numbers above the bands represent the relative expression of cleaved-caspase3 and cleaved-PARP1. The numbers above the bands represent the ratio of cleaved protein to total protein, respectively. **(F)** Images showing FITC-Annexin V/PI flow cytometry in HCT116-derived cell lines. The regions corresponding to Q2 and Q3 are indicative of apoptotic cells.

To evaluate the role of p53 in cisplatin resistance, we first detected p53 expression by western blot in p53^+/+^ and p53^−/−^ HCT116 cells. p53 was undetectable in HCT116 p53^−/−^ cells (Figure 1A). The CCK-8 assay results showed that, compared to that in HCT116 p53^+/+^, p53 deficiency attenuated the growth inhibition effect of cisplatin (Figure 1B). The clonogenic assay results showed that p53 deficiency significantly reduced the colony formation ability of HCT116 cells (Figure 1C). qPCR results showed that HCT116 p53^−/−^ cells highly expressed cisplatin-resistance related genes including ATP7A, ATP7B and MUC16 than HCT116 p53^+/+^ cells (Figure 1D). Western blot showed that, compared with those in HCT116 p53^+/+^ cells, the expression levels of apoptosis-related proteins cleaved PARP1 and cleaved caspase3 were decreased in HCT116 p53^−/−^ cells under cisplatin treatment conditions (Figure 1E). Flow cytometry further confirmed an increase in the number of apoptotic cells among the cisplatin-treated p53^−/−^ HCT116 cells (Figure 1F). Together, the above data suggested that p53 deficiency enhanced the cytotoxic tolerance of HCT116 cells to cisplatin treatment.

### Global landscape of the p53-regulated crotonylome in HCT116 cells

**FIGURE 2:**
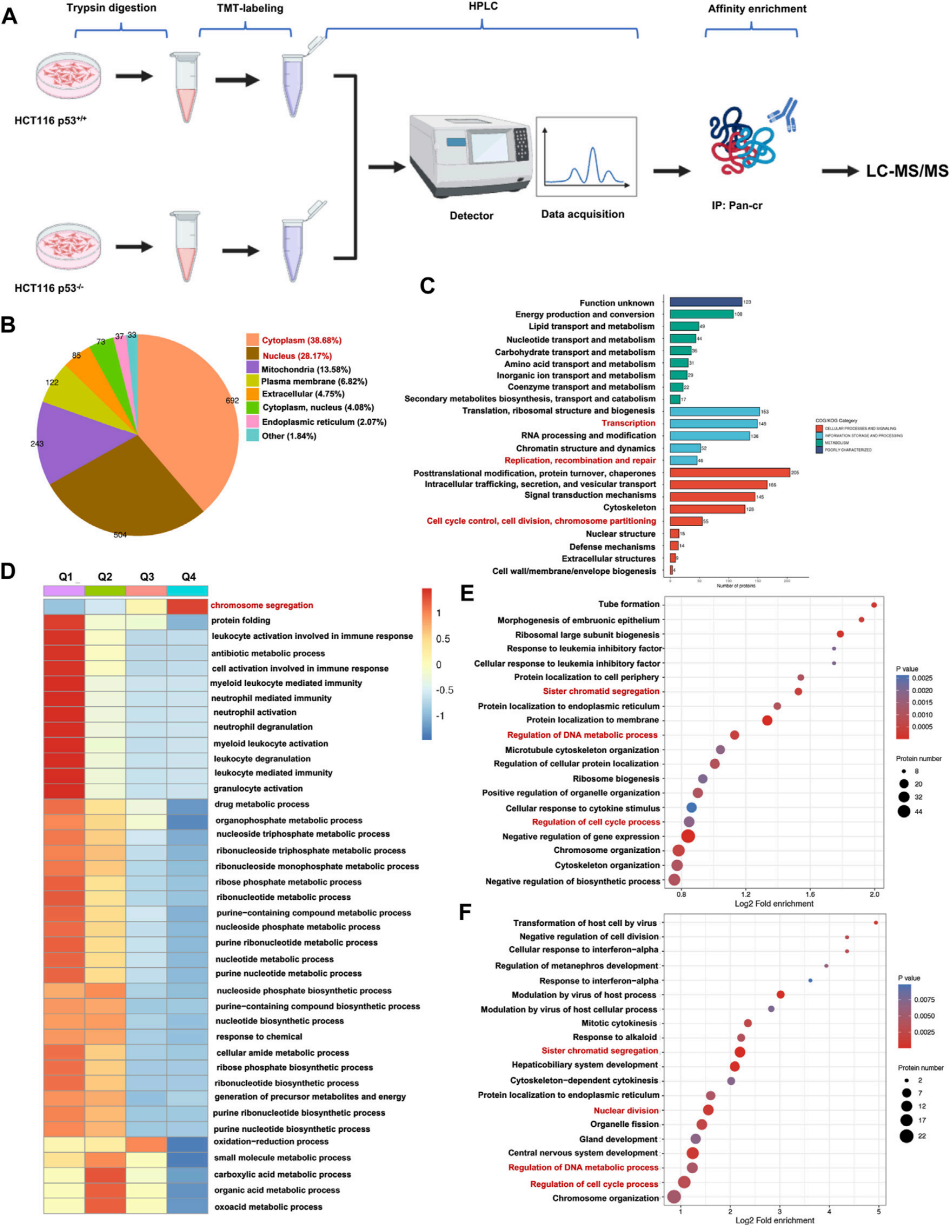
Global landscape of the p53-regulated crotonylome in HCT116 cells. **(A)** A schematic diagram illustrating the workflow for TMT-labeled LC-MS/MS analysis of Kcr proteins in both HCT116 p53^+/+^ and HCT116 p53^−/−^ cells. **(B)** Pie chart depicting the subcellular distribution of Kcr proteins with statistically significant variations. **(C)** Bar graphs showing the COG/KOG enrichment analysis of Kcr proteins. **(D)** Heatmap showing that cluster enrichment analysis of Kcr proteins. **(E, F)** Bubble charts were used to present genes in Q3 **(E)** and Q4 **(F)** clusters enrichment analysis.

To obtain a comprehensive and quantitative analysis of p53-related nonhistone Kcr, we conducted crotonylomic sequencing and analysis. A series of methods, such as trypsin digestion, TMT labeling, HPLC fractionation, affinity enrichment and LC‒MS/MS, were used to investigate Kcr substrates in HCT116 p53^+/+^ and HCT116 p53^−/−^ cells (Figure 2A). We utilized Wolfpsort (https://wolfpsort.hgc.jp) to predict the subcellular localization of proteins with significantly altered crotonylation sites. Our analysis revealed that 38.68% and 28.17% of the Kcr proteins were localized in the cytoplasm and nucleus, respectively (Figure 2B). Next, we conducted a COG/KOG functional classification analysis of the Kcr proteins to investigate their functional categories. We found that the functions of Kcr proteins regulated by p53 were predominantly enriched in the cell cycle, DNA replication, transcription and repair (Figure 2C). Based on the differential expression of p53 knockout-associated Kcr, we further divided the genes into four groups: Q1 (<0.5), Q2 (0.5-0.667), Q3 (1.5-2), and Q4 (>2.0). Then, for each Q group, we conducted GO classification, KEGG pathway, and protein domain enrichment analysis, followed by clustering analysis, aiming to identify the relevance of protein functions with different differential expression multiples in the comparison group. To our surprise, we found that the p53-upregulated crotonylated proteins (Q3 and Q4 groups) were predominantly associated with chromosome segregation (Figure 2D). Therefore, to investigate the specific functional classification of the upregulated crotonylated proteins, we conducted enrichment analysis for all the upregulated crotonylated proteins in Q3 and Q4. We found that, whether in the Q3 or Q4 group, the majority of crotonylated proteins were functionally concentrated in the cell cycle, DNA replication, transcription, and DNA metabolism processes (highlighted in red, Figures 2E, F). Therefore, we focused on the upregulated croyonylated protein groups involved in various biological processes related to DNA. We identified the top 20 significantly p53-upregulated crotonylated proteins for analysis. Among them, we identified the RRM2 protein, which plays a crucial role in DNA synthesis.

### p53 deficiency contributes to cisplatin resistance by upregulating RRM2 expression

**FIGURE 3:**
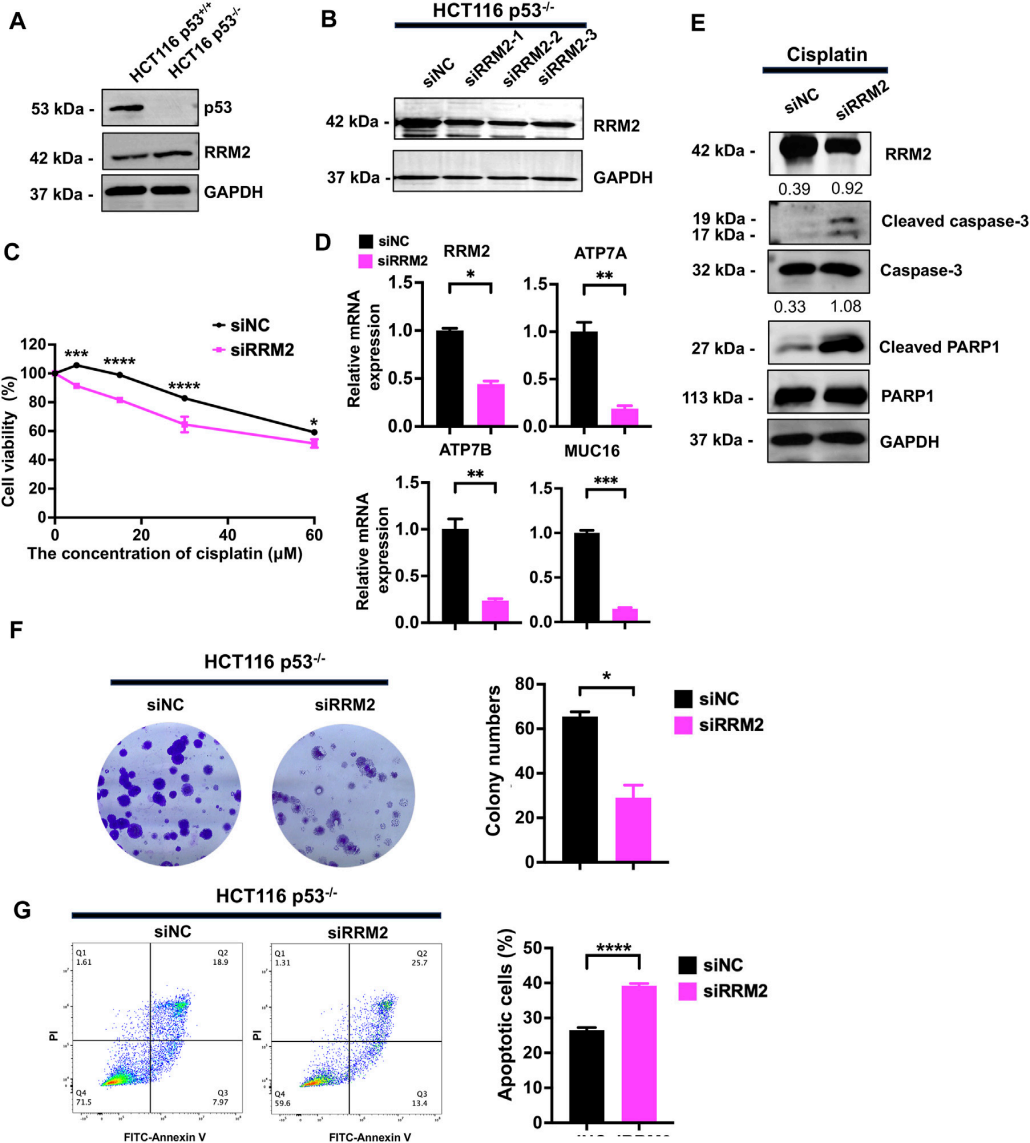
Knockdown of RRM2 in HCT116 p53^−/−^ resulted in sensitivity to cisplatin. **(A)** RRM2 expression level was detected by western blot in HCT116 p53^+/+^ and HCT116 p53^−/−^ cells. **(B)** The knock-down efficiency of three strands of siRRM2 in HCT116 p53^−/−^ cells was examined by western blot. siNC was used as a negative control. **(C)** Transfected HCT116 p53^−/−^ cells with siNC and siRRM2 were treated with cisplatin at concentrations of 0, 5, 15, 30, and 60 μM, and cell viability was assessed using the CCK-8 assay. **(D, E)** Transfected HCT116 p53^−/−^ cells with siNC and RRM2 for 48 h, then treated with 30 μM cisplatin for 48 h, and detected the expression of RRM2, ATP7A, ATP7B and MUC16 by qPCR **(D)** and RRM2, cleaved-PARP1 and cleaved-caspase3 by western blot. GAPDH, caspase3 and PARP1 sever as internal controls of RRM2, cleaved-caspase3 and cleaved-PARP1, respectively. The numbers above the bands represent the relative expression of cleaved-caspase3 and cleaved-PARP1 **(E)**. **(F)** HCT116 p53^−/−^ cells were transfected with siNC and siRRM2 for 48 h. The colony formation assay was performed on the cells treated with the drugs for a period of 14 days. **(G)** FITC-Annexin V/PI dual staining assay was used to detect the effect of RRM2 on apoptosis induced by cisplatin in HCT116 p53^−/−^ cells.

To determine whether RRM2 is involved in the cisplatin resistance induced by p53 knockout, we overexpressed and knocked down RRM2 in p53^+/+^ and p53^−/−^ HCT116 cells, respectively. We first detected the protein expression of RRM2, and its expression was upregulated in p53^−/−^ HCT116 cells (Figure 3A). We then knocked down RRM2 expression by transfecting siRNAs specifically targeting RRM2 into p53^−/−^ HCT116 cells. The efficiency was determined, and we selected the second siRNA strand for further experiments (Figure 3B). A CCK-8 assay showed that knockdown of RRM2 decreased the viability of cells treated with cisplatin (Figure 3C). qPCR results showed that knockdown of RRM2 reduced cisplatin-resistance related genes ATP7A, ATP7B and MUC16 expression (Figure 3D). Western blot analysis demonstrated that knock-down of RRM2 markedly increased the expression levels of the cleaved-PARP1 and cleaved-caspase3 proteins (Figure 3E). Consistently, the colony formation ability decreased after RRM2 knockdown in the presence of cisplatin (Figure 3F). Flow cytometry confirmed that the number of apoptotic cells was significantly increased when RRM2 was knocked down in p53^−/−^ HCT116 cells (Figure 3G).

**FIGURE 4:**
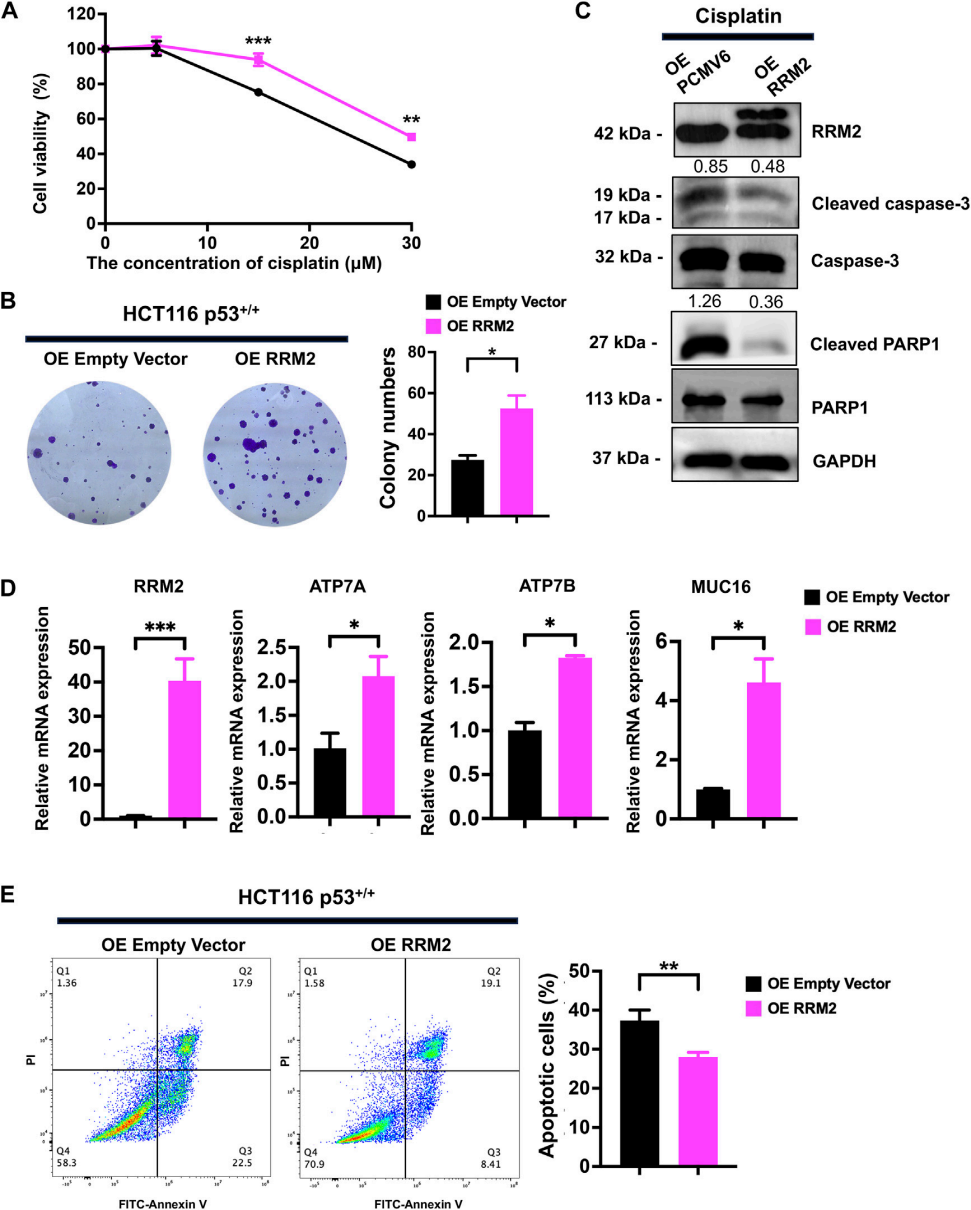
Overexpression of RRM2 in HCT116 p53^+/+^ resulted in cisplatin resistance. **(A)** The CCK-8 assay was performed to assess the cell viability of HCT116 p53^+/+^ cells (transfected with an Empty Vector and RRM2 plasmid) following treatment with 0, 5, 15, and 30 μM cisplatin. **(B)** HCT116 p53^+/+^ cells were transfected with Empty vector and RRM2 plasmids for 48 h. Subsequently, the colony formation assay was conducted on the cells treated with cisplatin for a duration of 14 days. **(C)** The western blots showed the expression of RRM2, cleaved-PARP1 and cleaved-caspase3 following treatment with a concentration of 25 μM cisplatin in HCT116 p53^+/+^ cells transfected with Empty Vector and RRM2 plasmids. The numbers above the bands represent the relative expression of cleaved-caspase3 and cleaved-PARP1. The numbers above the bands represent the ratio of cleaved protein to total protein, respectively. **(D)** The qPCR showed the expression of RRM2, ATP7A, ATP7B and MUC16 following treatment with a concentration of 30 μM cisplatin in HCT116 p53^+/+^ cells transfected with Empty Vector and RRM2 plasmids. **(E)** Annexin V/PI dual staining assay detection of apoptosis in RRM2 overexpression and control of HCT116 p53^+/+^ cells with cisplatin treatment.

In HCT116 p53^+/+^ cells, we overexpressed RRM2 in a rescue assay to confirm the contribution of RRM2 to cisplatin resistance. As expected, RRM2 overexpression enhanced HCT116 p53^+/+^ cell viability (Figure 4A) and colony formation ability (Figure 4B) under cisplatin treatment. Meanwhile, RRM2 overexpression decreased cleaved-PARP1 and cleaved-caspase3 expression at protein level (Figure 4C), and increased ATP7A, ATP7B and MUC16 expression at mRNA level (Figure 4D). Flow cytometry assays also showed that HCT116 p53^+/+^ cells overexpressing RRM2 underwent less apoptosis (Figure 4E). Taken together, these data indicated that p53 deficiency contributed to cisplatin resistance through RRM2 overexpression.

### RRM2^K283Cr^ upregulation promotes cisplatin resistance in HCT116 p53^−/−^ cells

**FIGURE 5:**
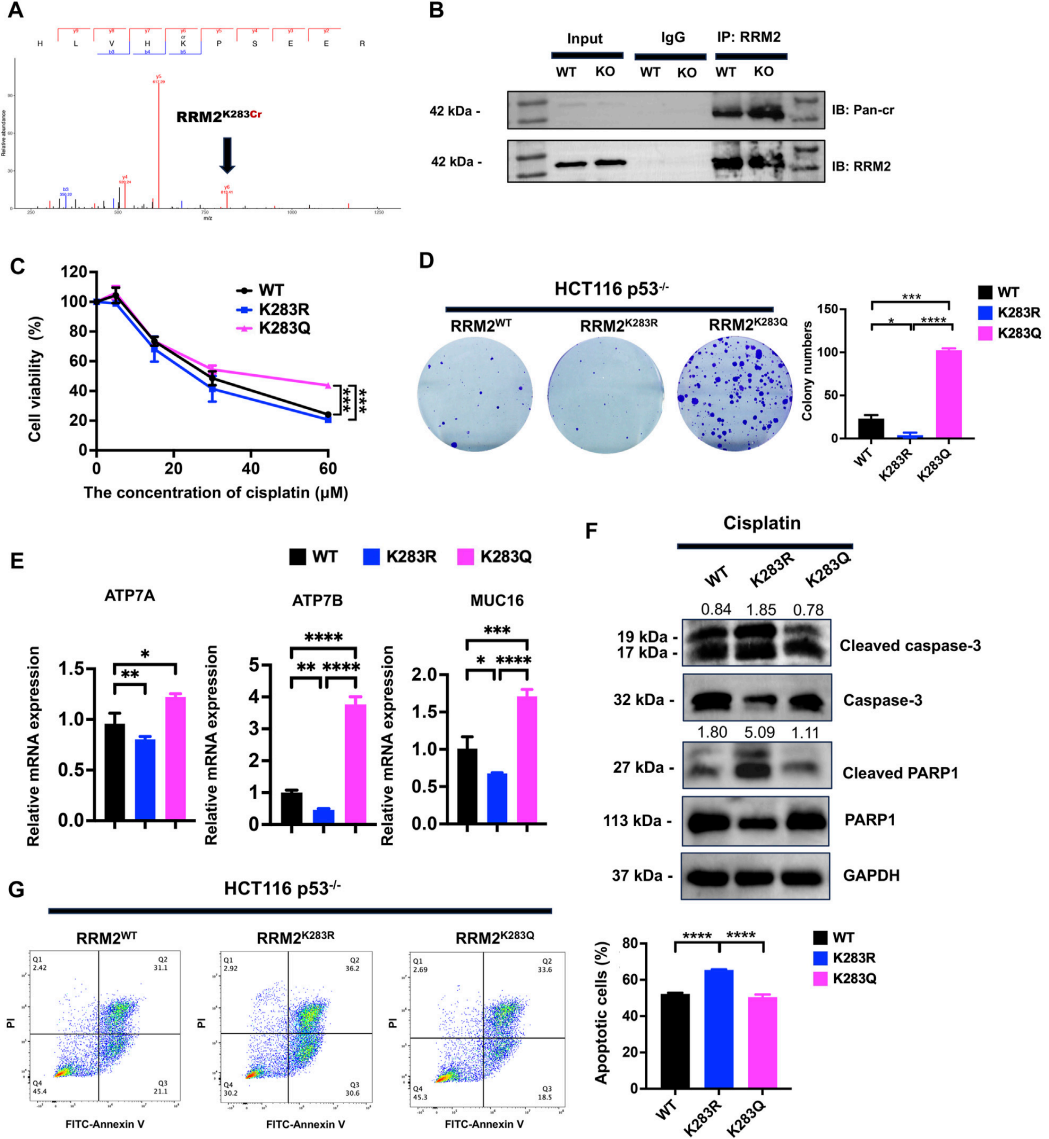
RRM2^K283Cr^ promotes the resistance of HCT116 cells to cisplatin. **(A)** RRM2^K283Cr^ in HCT116 p53^−/−^ cells by mass spectrometry. **(B)** Co-IP using anti-RRM2 antibody, followed by immunoblotting with anti-Kcr and anti-RRM2 antibodies in both HCT116 p53^+/+^ and HCT116 p53^−/−^ cells. **(C)** The RRM2^WT^, RRM2^K283R^, and RRM2^K283Q^ expression plasmids were constructed and individually transfected into HCT116 p53^−/−^ cells for 48 h. Subsequently, a CCK-8 assay was conducted to assess cell viability at concentrations of 0, 5, 15, 30, and 60 μM. **(D)** A colony formation assay was performed to evaluate HCT116 p53^+/+^ cell proliferation under the condition of 10 μM cisplatin. **(E)** The qPCR showed the expression of ATP7A, ATP7B and MUC16 following treatment with a concentration of 30 μM cisplatin in HCT116 p53^−/−^ cells transfected with RRM2^WT^, RRM2^K283R^, RRM2^K283Q^ plasmids. **(F)** Cleaved-PARP1, cleaved-caspase3 and GAPDH expression level were detected by western blot in HCT116 p53^−/−^ cells transfected with RRM2^WT^, RRM2^K283R^, RRM2^K283Q^ plasmids with the treatment of 30 μM cisplatin. The numbers above the bands represent the relative expression of cleaved-caspase3 and cleaved-PARP1. Caspase3 and PARP1 served as cleaved-caspase3 and cleaved-PARP1 internal controls, respectively. **(G)** FITC-Annexin V/PI dual staining assay was performed to detect the apoptotic cells induced by cisplatin.

The mass spectrogram of RRM2^K283Cr^ clearly showed that RRM2 could be highly crotonylated at the K283 site in p53^−/−^ HCT116 cells (Figure 5A). To confirm the LC‒MS/MS results, a co-IP assay was performed with the same volume of anti-RRM2 antibody. The results showed that crotonylation at K283 in RRM2 was significantly increased in p53^−/−^ HCT116 cells (Figure 5B). To explore the effect of RRM2^K283Cr^ on cisplatin resistance in p53^−/−^ HCT116 cells, we first constructed an RRM2^K283Cr^ inactivation plasmid (RRM2^K283R^) and an RRM2^K283Q^ activation plasmid (RRM2^K283Q^) and transfected them into p53^−/−^ HCT116 cells. Compared with RRM2^K283R^ cells, p53^−/−^ HCT116 cells transfected with RRM2^K283Q^ were confirmed to be cisplatin resistance by CCK-8 and colony formation assays (Figure 5C, D). qPCR results showed the increased mRNA expression of ATP7A, ATP7B and MUC16 in RRM2^K283Q^ transfected HCT116 p53^−/−^ cells than those in RRM2^K283R^ transfected HCT116 p53^−/−^ cells (Figure 5E). Western blot analysis revealed decreased levels of cleaved PARP1 and cleaved caspase three in HCT116 p53^−/−^ cells transfected with the RRM2^K283Q^ plasmid compared to those in cells transfected with the RRM2^K283R^ plasmid (Figure 5F). Meanwhile, the number of apoptotic RRM2^K283R^ cells increased, but RRM2^K283Q^ decreased cell apoptosis after cisplatin treatment (Figure 5G). Taken together, these findings indicate that increased crotonylation at the RRM2^K283^ site could enhance the cytotoxic tolerance of HCT116 cells to cisplatin treatment after p53 knockout.

### SIRT7 decreased the crotonylation of RRM2^K283Cr^

**FIGURE 6:**
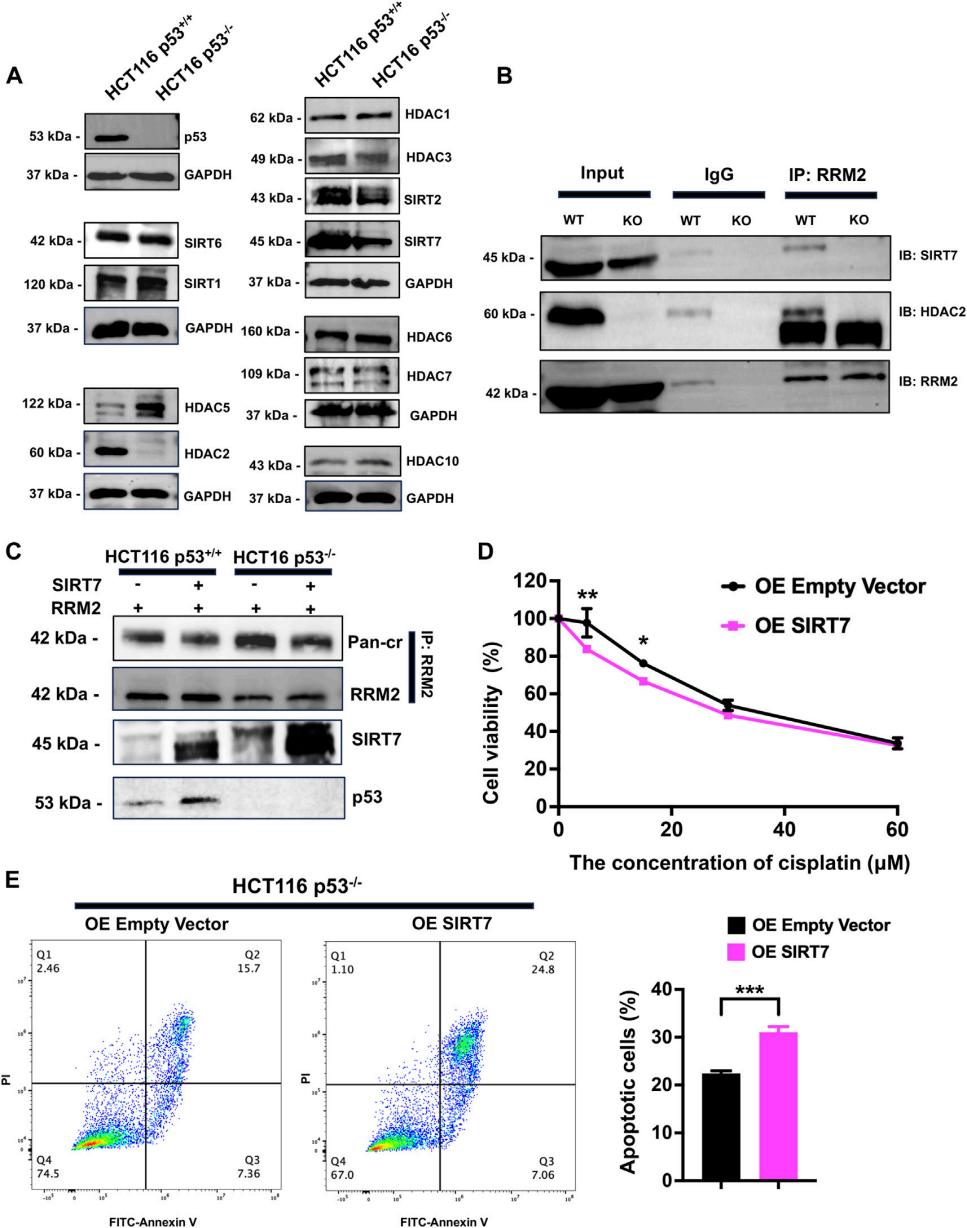
SIRT7 serves as a decrotonylase for RRM2, and overexpression of SIRT7 in HCT116^−/−^ increases the sensitivity of cells to cisplatin. **(A)** The western blot was used to measure the expression levels of Zn^2+^-dependent HDACs and NAD^+^-dependent sirtuins in HCT116 p53^+/+^ and HCT116 p53^−/−^ cells. **(B)** Co-IP experiments were conducted in HCT116 p53^+/+^ and HCT116 p53^−/−^ cells using anti-RRM2 and anti-IgG, followed by immunoblotting with anti-HDAC2 and anti-SIRT7. **(C)** Co-IP was carried out to observe the crotonylation of RRM2 with or without overexpression of SIRT7 both in HCT116 p53^+/+^ and HCT116 p53^−/−^ cells. **(D, E)** HCT116 p53^+/+^ cells were transfected with SIRT7 over-expression plasmid for 48 h. CCK-8 **(D)** and flow cytometry **(E)** assays were conducted to detected the cisplatin resistance ability with the treatment of cisplatin.

To explore the decrotonylation of RRM2^K283Cr^ in p53^−/−^ HCT116 cells, we examined the expression of lysine decrotonylases, such as HDACs and SIRTs. Western blot analysis revealed that HDAC2 and SIRT7 were dramatically decreased in p53^−/−^ HCT116 cells (Figure 6A). Given the highly increased crotonylation of RRM2 in p53^−/−^ HCT116 cells, we preferentially selected decrotonylases with low expression upon p53 knockout for further exploration. We therefore paid attention to SIRT7 because the expression of SIRT7 was dramatically decreased in p53^−/−^ HCT116 cells. Next, co-IP was performed to verify the decrotonylase role of SIRT7 for RRM2^K283Cr^. Total cellular extracts from HCT116 p53^+/+^ and HCT116 p53^−/−^ cells were immunoprecipitated, and the results showed that RRM2 could interact with SIRT7 (Figure 6B). To further confirmed that SIRT7 is a decrotonylase of RRM2^K283Cr^, we overexpressed SIRT7 in both HCT116 p53^+/+^ and HCT116 p53^−/−^ cells and used a co-IP assay to detect the interaction between SIRT7 and RRM2. The results indicated that the overexpression of SIRT7 reduced the Pan-cr expression level (Figure 6C). Then, we used a CCK-8 assay to confirm cell viability after treatment with cisplatin in p53^−/−^ HCT116 cells transfected with a SIRT7 overexpression plasmid. As anticipated, cells transfected with the SIRT7 plasmid exhibited a diminished ability to resist cisplatin (Figure 6D). Flow cytometry again demonstrated an increased proportion of apoptotic HCT116 p53^−/−^ cells transfected with the SIRT7 overexpression plasmid after cisplatin treatment (Figure 6E), which revealed that the cells with decreased RRM2^K283Cr^ caused by SIRT7 overexpression were more sensitive to cisplatin. Overall, p53 decreased the expression of SIRT7, which acts as a decrotonylase for RRM2, enhancing RRM2^K283Cr^ and consequently increasing the sensitivity of HCT116 cells to cisplatin.

### p53 deficiency mediates cisplatin resistance by increasing RRM2 protein expression and RRM2^K283Cr^ by decreasing SIRT7 expression *in vivo*

**FIGURE 7:**
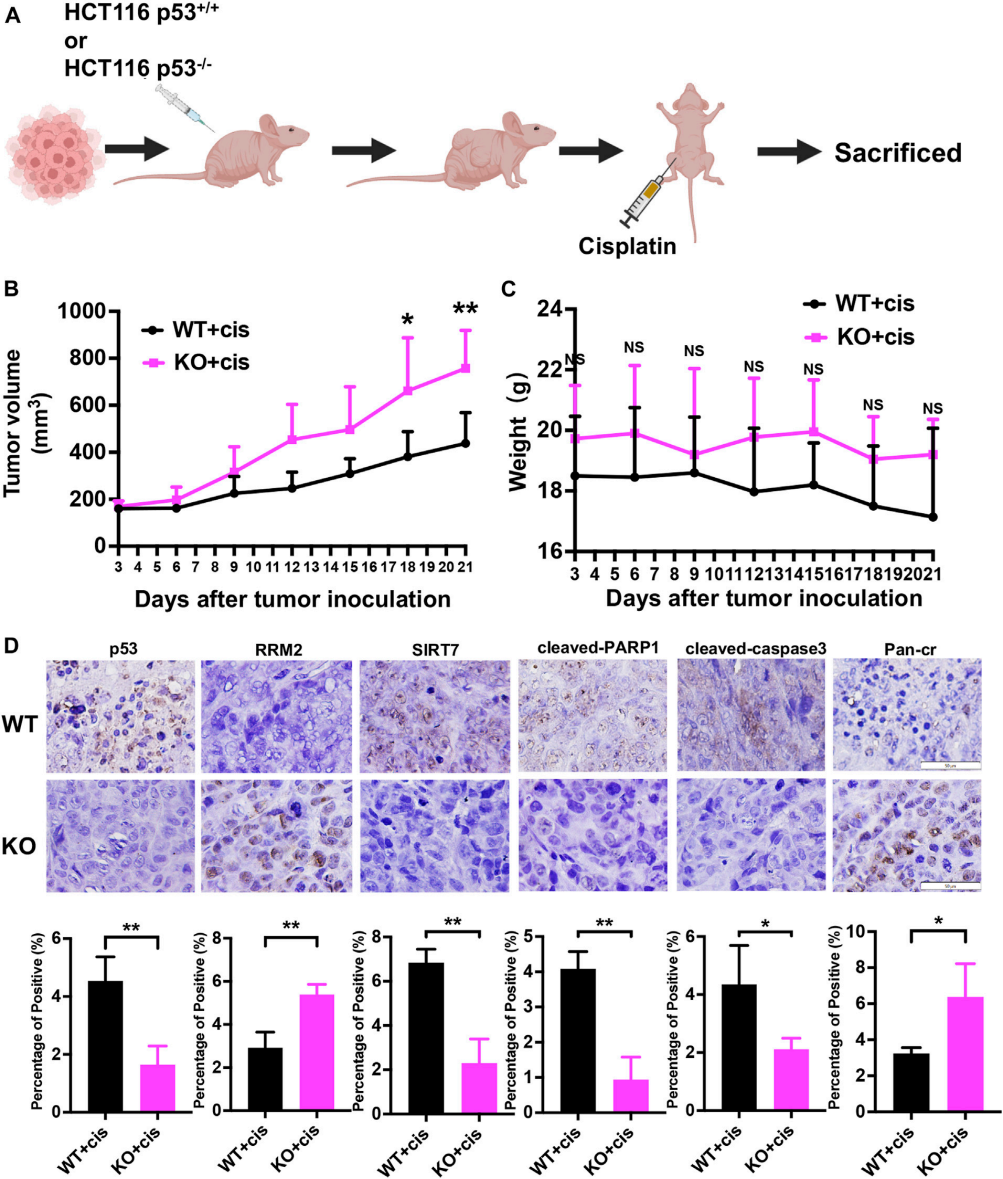
p53 deficiency leads to cisplatin resistance by upregulating RRM2 protein expression and RRM2^K283Cr^, while downregulating SIRT7 expression *in vivo*. **(A)** Subcutaneous xenograft assay of HCT116 p53^+/+^ and HCT116 p53^−/−^ cells treated with cisplatin. **(B)** The volumes of the tumors were measured every 3 days after tumor inoculation. **(C)** The weight of mice was monitored at the specified time points. **(D)** Representative images for immunostaining of p53, RRM2, SIRT7, cleaved-PARP1, cleaved-caspase3 and Pan-Cr in subcutaneous xenograft tumors.

Given the crucial role of both RRM2 expression and RRM2^K283Cr^ in colon cancer cells after p53 deficiency *in vitro*, to investigate whether their expression *in vivo* mirrors that observed *in vitro*, we performed subcutaneous xenograft and cisplatin treatment. HCT116 p53^+/+^ and HCT116 p53^−/−^ cells were injected subcutaneously into nude mice, and cisplatin was injected intraperitoneally after tumor formation (Figure 7A). As expected, the HCT116 p53^−/−^ (KO) group exhibited greater resistance to cisplatin than the wild-type (WT) group (Figure 7B). However, there was no significant difference in body weight between the two groups of mice (Figure 7C). Immunostaining of the xenograft tumor sections revealed that, in comparison to the WT + cis group, the KO + cis group exhibited significant downregulation of SIRT7, cleaved-PARP1 and cleaved-caspase3 expression and substantial upregulation of RRM2 and Pan-cr expression, which is in line with our *in vitro* findings (Figure 7D).

## Discussion

In this study, we first proposed that RRM2 is involved in the regulation of p53-mediated cisplatin resistance. Our data revealed a novel mechanism of p53 deficiency-induced cisplatin resistance mediated through the p53/SIRT7/RRM2^K283Cr^/cleaved-PARP1 and cleaved-caspase3 axes, providing a rationale for overcoming p53 mutant/deficiency-driven chemoresistance in colon cancer cells.

In more than 50% of human cancers, the p53 gene is mutated, while the remaining cases involve the biological inactivation of its pathway (Lain and Lane, 2003). This inactivation includes MDM2 amplification (Freedman et al., 1999), loss of p14^ARF^ (Sherr, 1998), and mutations in activating kinase such as ATM(23). The loss of p53 pathway function gives cancer cells a survival advantage over the toxicity of chemotherapeutic agents, enabling them to evade the resolution of oncogenic signals and DNA damage, thereby sustaining abnormal proliferation. In addition, in some cancers, the intact p53 gene is mutated by multiple mechanisms, leading to resistance to chemotherapy drugs. Despite numerous studies that have clarified the role and mechanisms of p53 in chemotherapy resistance, effective targets for p53 mutation are still lacking. Therefore, there is an urgent need for effective targets to reverse the chemotherapy resistance caused by the loss of p53 function in these tumors. Here, we found that cisplatin resistance is promoted by p53 deficiency in HCT116 cells. Consistent with the *in vitro* experiments, subcutaneous xenograft and cisplatin treatment showed that nude mice harboring HCT116 p53^−/−^ cells developed larger tumors than those harboring HCT116 p53^+/+^ cells with the same cisplatin administration.

These findings lead us to explore the potential mechanisms of cisplatin resistance caused by p53 deficiency to identify a new target to improve the prognosis of patients with p53 mutation/deficiency tumors. Therefore, we identified RRM2 in the p53-upregulated crotonylation data. Previous studies have shown that, as the only small subunit of the enzyme complex RNR that catalyzes *de novo* synthesis of deoxyribonucleic acid (DNA), RRM2 provides deoxyribonucleotide triphosphates (dNTPs) for DNA replication and repair in proliferating cells during the S phase of the cell cycle. Through crotonylation and experimental validation, we found that both RRM2 expression and K283 crotonylation are significantly upregulated after p53 deficiency. Accordingly, we knocked down and overexpressed RRM2 in HCT116 p53^−/−^ and HCT116 p53^+/+^ cells, respectively, to demonstrate the role of RRM2 in cisplatin resistance. These results were consistent with the conclusions of previous studies (Itoi et al., 2007; Zhan et al., 2021). In many cancers, RRM2 overexpression is correlated with low chemotherapy sensitivity and poor prognosis (Dasika et al., 1999; Itoi et al., 2007). However, the impact of posttranslational modifications to RRM2, especially the crotonylation of the RRM2 protein, on tumorigenesis remains elusive. Therefore, we constructed a mutant plasmid for the RRM2^K283^ site to further study the effect of RRM2^K283Cr^ on cisplatin resistance. Importantly, we found that upregulated RRM2^K283Cr^ inhibited cell apoptosis by suppressing the expression of the cisplatin resistance genes ATP7A, ATP7B (Petruzzelli and Polishchuk, 2019) and MUC16 (Boivin et al., 2009) and the apoptosis-related proteins cleaved-PARP1 and cleaved-caspase3 (Xu et al., 2023), thereby increasing cell resistance to cisplatin after p53 knockout. Our study extends the understanding of the function of RRM2^K283Cr^ in cisplatin resistance in colon cancer and identifies a target for cisplatin resistance caused by loss of p53 pathway function.

Furthermore, in our extensive search for the cause of the elevated RRM2^K283Cr^, we found that the protein expression of SIRT7 decreased after p53 knockout. Subsequently, we verified the ability of SIRT7 to bind to RRM2 via co-IP experiments, confirming that SIRT7 is the decrotonylase of RRM2. In addition, we also found that the overexpression of SIRT7 in HCT116 p53^−/−^ cells rescued cisplatin resistance. Recently, increasing evidence has indicated that SIRT7 expression is altered in various human cancers, indicating its significant and controversial roles in tumor biology. For instance, Yu et al. demonstrated that SIRT7 overexpression exhibits oncogenic effects and serves as a prognostic factor in colorectal cancer (Yu et al., 2014). Additionally, SIRT7 interacts with LAP2α in breast cancer, and inhibition of the SIRT7/LAP2α axis represents a potential therapeutic strategy for preventing breast cancer metastasis (Huo et al., 2023). SIRT7 is an NAD^+^-dependent deacetylase that targets H3K18Ac, stabilizing the transformed state of cancer cells. This finding underscores the pivotal role of SIRT7 in chromatin regulation, cellular transformation programs, and tumor formation (Barber et al., 2012). Although most studies have shown that high SIRT7 expression is positively correlated with tumor migration, invasion, and proliferation, the effect of SIRT7 on chemotherapy resistance is still unknown. Su et al. reported USP17L2 knowdown-mediated SIRT7 polyubiquitination led to breast cancer cells sensitizing to chemotherapy, which suggests that SIRT7 acts as a procancer protein (Su et al., 2022). To date, these findings suggest that SIRT7 is a potential therapeutic target for tumor therapy.

Before our study, there are only three papers regarding crotonylation and colon cancer. We have cited them in the introduction section. Hou et al. reported ENO1 regulates colorectal cancer through crotonylation (Hou et al., 2021a). Liao M et al. reported the relationship between histone H3K27 crotonylation and DNA damage of colon cancer (Liao et al., 2022). Qu M et al. revealed a novel mechanism of histone crotonylation-mediated ATX expression regulation by in a HIF-2α-dependent manner in a colon cancer SW480 cells (Qu et al., 2023). Compared with the three researches, we find a new crotonylated protein RRM2, which K283 site crontonylation contributes to cisplatin resistance. To data, there are only a few studies reported the function of non-histone protein crotonylation, our research finds a new crotonylated protein RRM2 and identify its pro-cancer role in colon cancer. This is our contribution to this field.

**FIGURE 8:**
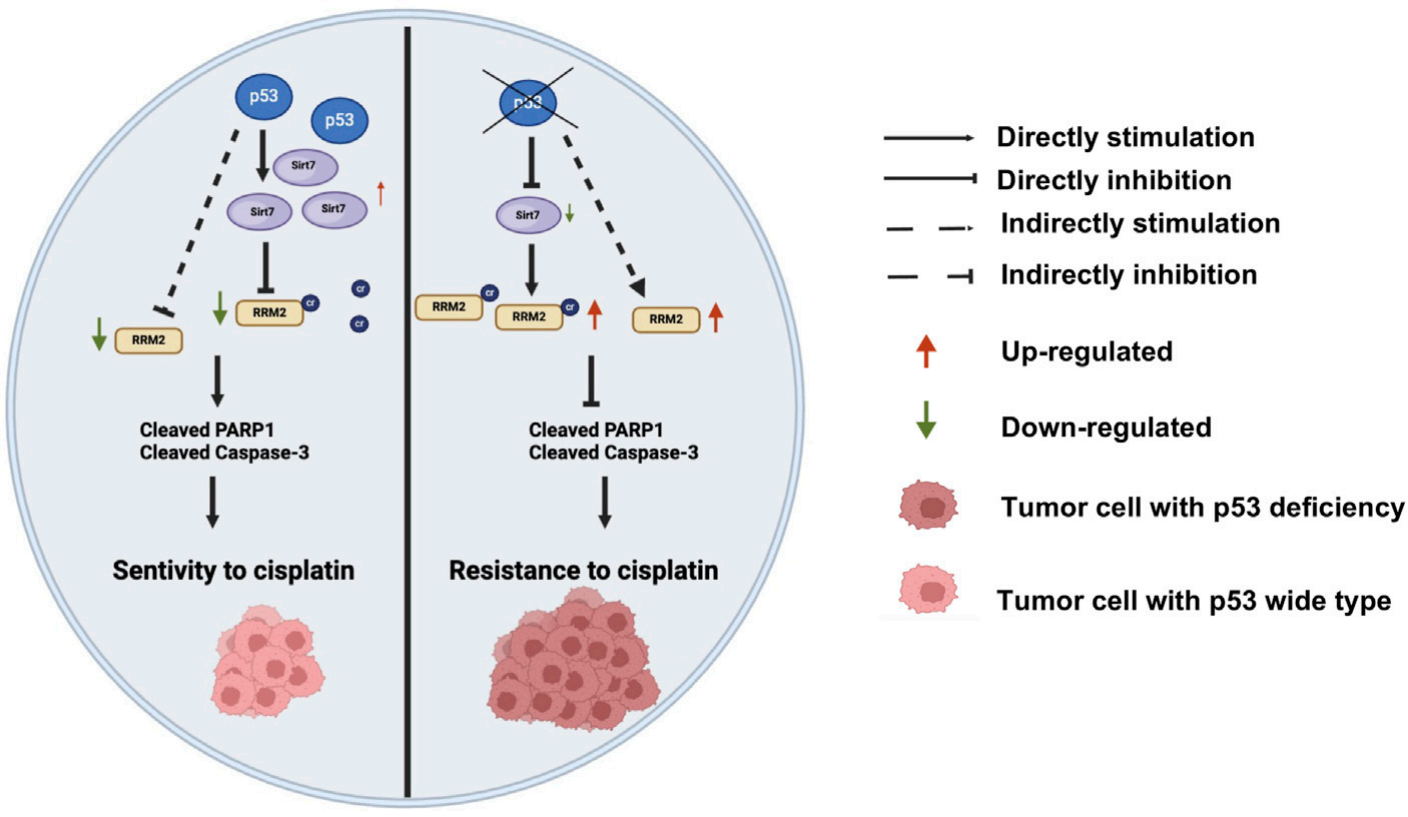
A novel mechanism of p53 knockout regulating cisplatin chemotherapy resistance through SIRT7/RRM2^K283Cr^/cleaved-PARP1 and cleaved-caspase3 axis-mediated apoptosis reduction in colon cancer. Additionally, p53 deficiency indirectly regulates the apoptosis-related proteins cleaved-PARP1 and cleaved-caspase3 by upregulating the expression of RRM2^K283Cr^ protein, thereby mediating cisplatin resistance.

In conclusion, we identified a molecular mechanism by which p53 deficiency induces cisplatin resistance through the p53/SIRT7/RRM2^K283Cr^ axis in colon cancer (Figure 8). As far as we know, this is the first study to verify the role of p53 deficiency-mediated RRM2^K238^ crotonylation in cisplatin-resistance of colon cancer, which may provide a new target for colon cancer treatment. We deduce nonhistone protein crotonylation posttranslation may play a vital role in the colon cancer development and treatment. Furthermore, given that some histone deacetylase inhibitors have been employed in anti-cancer treatment, our findings suggest a reassessment of the benefits and risks of these inhibitors treatment approach. However, the regulatory relationship between p53 and SIRT7 was not further explored in this study. The associations between RRM2 protein and RRM^K283Cr^, including DNA transcription, stress regulation, and the cell cycle, should be investigated in the future.

## Data Availability

The datasets presented in this study can be found in online repositories. The names of the repository/repositories and accession number(s) can be found below: PRIDE repository, https://www.ebi.ac.uk/pride/archive/projects/PXD047258.
